# Efficient Generation of *Myostatin* Gene Mutated Rabbit by CRISPR/Cas9

**DOI:** 10.1038/srep25029

**Published:** 2016-04-26

**Authors:** Qingyan Lv, Lin Yuan, Jichao Deng, Mao Chen, Yong Wang, Jian Zeng, Zhanjun Li, Liangxue Lai

**Affiliations:** 1 Jilin Provincial Key Laboratory of Animal Embryo Engineering, Institute of Zoonosis, College of Veterinary Medicine, Jilin University, Changchun 130062, China; 2 CAS Key Laboratory of Regenerative Biology, South China Institute for Stem Cell Biology and Regenerative Medicine, Guangzhou Institutes of Biomedicine and Health, Chinese Academy of Sciences, Guangzhou 510530.

## Abstract

CRISPR/Cas9 has been widely used in generating site-specific genetically modified animal models. *Myostatin (MSTN)* is a negative regulator of muscle mass, related to muscle growth and differentiation. The knockout of *MSTN* with the desired phenotype of double muscle has been successfully generated in mice, goats, pigs and cattle, but not in rabbits. In this study, the *MSTN* knockout (KO) rabbits were generated by co-injection of Cas9 mRNA and sgRNA into zygotes. The typical phenotype of double muscle with hyperplasia or hypertrophy of muscle fiber was observed in *MSTN* KO rabbits. Furthermore, a similar phenotype was found in the F1 generation, suggesting that the mutation of *MSTN* could be stably inherited in the *MSTN* KO rabbits. In summary, we have successfully generated *MSTN* KO rabbits using CRISPR/Cas9 system with high efficiency, which is a reliable and effective animal model for the study of muscle development and related diseases.

Gene editing technologies have been developed to study the function of genes, generate genetically modified animal models for biomedical research and improve animal traits in agriculture. CRISPR/Cas9 system consists of a single guide RNA (sgRNA) and a human codon-optimized Cas9 nuclease, which can induce targeted mutations by nonhomologous end joining (NHEJ). Co-injection of Cas9 mRNA and sgRNA into zygotes has been used as an efficient tool to generate gene-targeted animal models in mice^1^, sheep^2^, monkeys^3^ and pigs^4^.

Rabbits are a promising animal model for biomedical research, as they show more similarities to human beings in terms of physiology and anatomy than mice and rats, and require low cost maintenance and short pregnancy period compared to pigs and monkeys^5^. Currently, rabbits have been extensively used as a more appropriate animal model for studying cardiovascular/metabolic and ophthalmic diseases^6^,^7^.

*Myostatin* (*MSTN*) is a member of the transforming growth factor beta (TGF-β) superfamily, which acts as a negative regulator of muscle growth^8^,^9^. It has been reported that spontaneous mutations of *MSTN* in cattle^10^ and sheep^11^ causes muscle hypertrophy. A double-muscled phenotype with the characteristics of increased muscle mass was also obtained in *MSTN* KO sheep^2^, pigs^12^ and dogs^13^, which encourages us to generate *MSTN* KO rabbits for the study of muscle development and improvement of animal traits for agriculture in the future.

Here, in order to generate *MSTN* KO rabbits, the *in vitro* transcribed mRNA encoding for Cas9 and sgRNA targeting the *MSTN* gene, was microinjected into the cytoplasm of rabbit pronuclear-stage embryos. We demonstrated the high efficiency of CRISPR/Cas9 system-mediated gene editing and the desired phenotype of double muscle was obtained in the *MSTN* KO rabbits.

## Results

### CRISPR/Cas9-mediated *MSTN* KO in rabbit zygotes

**Figure 1:**
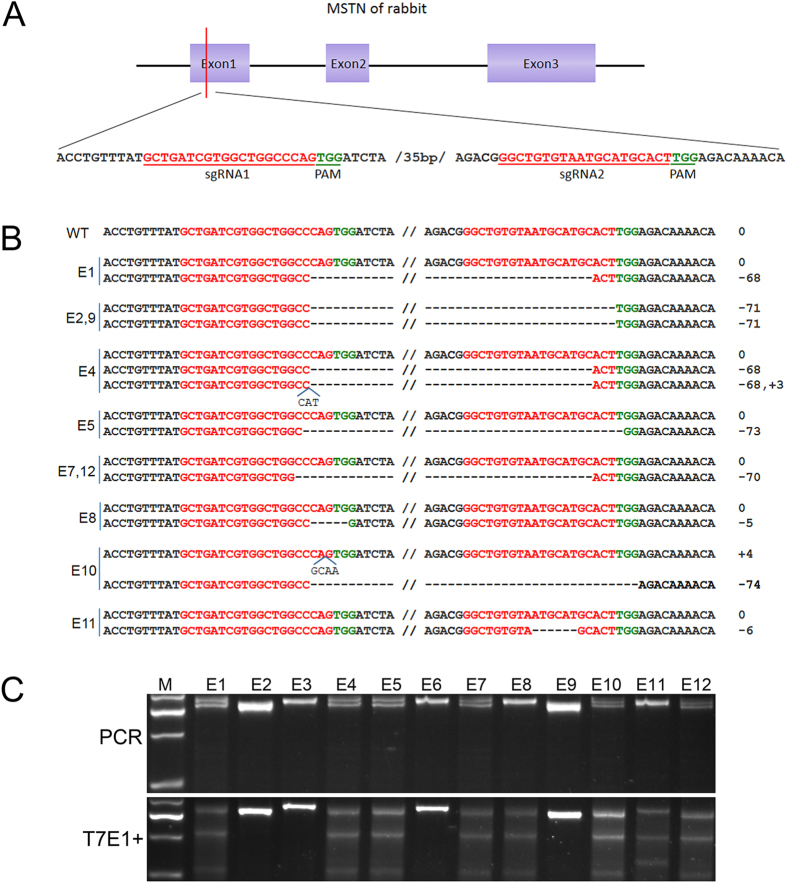
CRISPR/Cas9-mediated gene knockout of *MSTN* in zygote. (**A**) Schematic diagram of sgRNA targeting the rabbit *MSTN* gene loci. Two sgRNA sequences, sgRNA1 and sgRNA2, are marked in red and the protospacer adjacent moti (PAM) sequences are presented in green. (**B**) Mutation detection in blastocyst by T-cloning and Sanger sequencing. The WT sequence is shown at the top of the targeting sequence. E: embryos; WT: wild type; deletions “−”; insertion “+”. (**C**) Mutation detection in blastocyst by T7E1 cleavage assay. M, DL2000; 1–12 represent different blastocysts used in this study.

In order to disrupt the function of *MSTN* in rabbits, two sgRNAs targeting the CDS of the rabbit *MSTN* gene were designed using the CRISPR/Cas9 online design tool (http://tools.genomeengineering.org) (Fig. 1A). To determine the efficiency of CRISPR/Cas9 system in zygotes, the Cas9 mRNA and sgRNAs were microinjected into rabbit zygotes and cultured until the blastocyst stage. Then 12 blastocysts were harvested and subjected to PCR amplification and T7E1 cleavage assay. As shown in Fig. 1B,C, the mutation of *MSTN* was found in 10 tested blastocysts (83.3%), in which 3 blastocysts (25%) displayed biallelic mutations, suggesting that the two sgRNAs-directed CRISPR/Cas9 system was an efficient tool for disrupting the rabbit *MSTN* gene in zygotes.

### Generation of *MSTN* KO rabbits by CRISPR/Cas9 system

**Figure 2:**
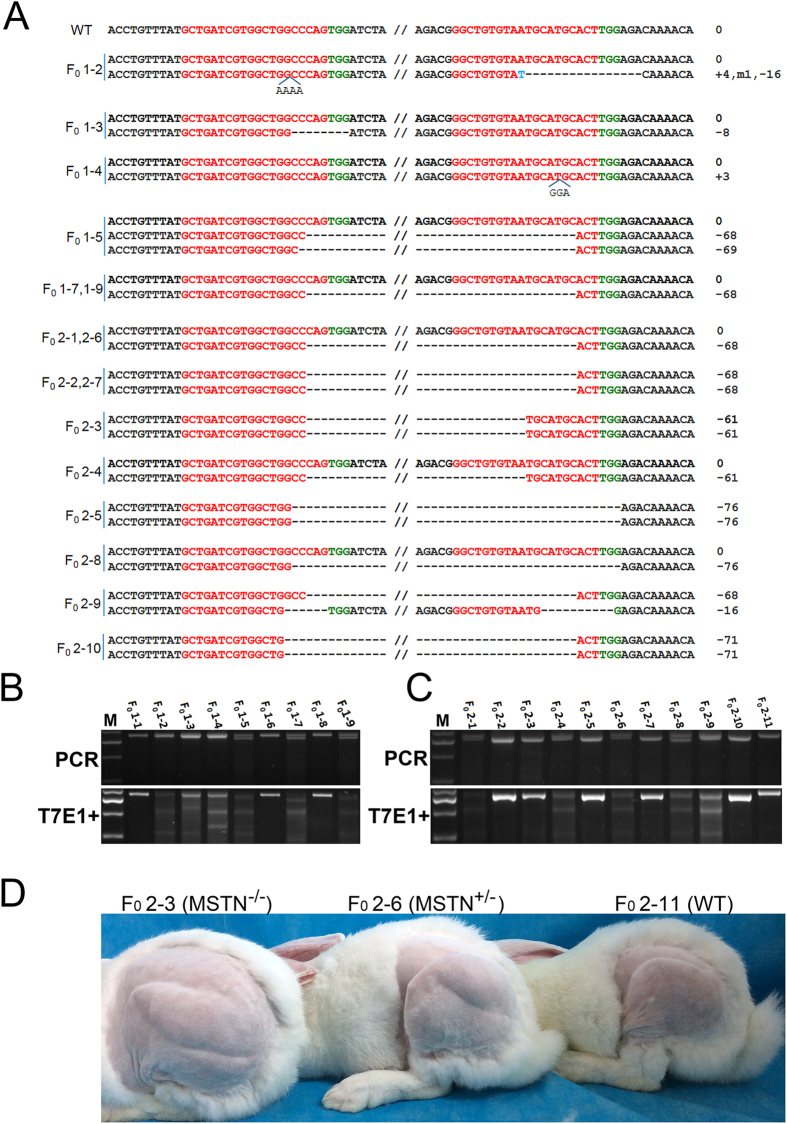
Generation of *MSTN* KO rabbit via zygote injection. (**A**) T-cloning and Sanger sequencing of 20 pups. F_0_1-1–F_0_1–9 represent the F0 pups from the first time of microinjection. F_0_2-1–F_0_2–11 represent the F0 pups from the second microinjection. The sequences of sgRNA are shown in red and the PAM sequences are presented in green. The length of deletions is noted to the right of each sequence (- deletion). WT: wild type; deletions “−”; insertion “+”. (**B**) T7E1 cleavage assay for the mutation detection of F_0_1-1–F_0_1–9. M, DL2000; 1–9 represent the pups used in this study. (**C**) T7E1 cleavage assay for the mutation detection of F_0_2-1–F_0_2–11. M, DL2000; 1–11 represent the pups used in this study. (**D**) Photos of *MSTN*^−/−^ (F_0_2–3), *MSTN*^+/−^ (F_0_2–6) and WT (F_0_2–11) rabbits (4-month old) in F0. Note that an obvious double muscular phenotype was found in *MSTN*^−/−^ and *MSTN*^+/−^ rabbits, which compared to the WT.

To generate *MSTN* KO rabbits, a total of 158 injected zygotes were transferred into the oviducts of 4 surrogate rabbits. After a full-term gestation, the 4 surrogate mothers gave birth to 20 live pups successfully (Table 1). The T-cloning and PCR-sequence results showed that the *MSTN* mutation was detected in 16 pups, and the indels ranged from 3 bp to 76 bp (Fig. 2A), which was also confirmed by T7E1 assay (Fig. 2B,C). In addition, fragment deletions between the two sgRNAs targeting sites were frequently observed in this study (81.3%). Furthermore, the typical phenotype of double muscle was observed in F0 *MSTN* KO rabbits at 4 months of age, which compared with their wild type (WT) counterparts (Fig. 2D).

Off-target effect is a major concern of the CRISPR/Cas9 system. To test whether off-target effect occurred in these genetically modified rabbits, a total of 5 potential off-target sites (POTS) for each sgRNA were predicted by the CRISPR design online tool. All POTS were PCR amplified and subjected to T7E1 cleavage and Sanger sequence. The results showed that no mutation was detected in these POTS, indicating that the Cas9/sgRNA system did not induce undesirable off-target effect in the *MSTN* KO rabbits (Supplementary Table S2 and Supplementary Fig. S1).

### Heritability of the *MSTN* KO rabbits

**Figure 3:**
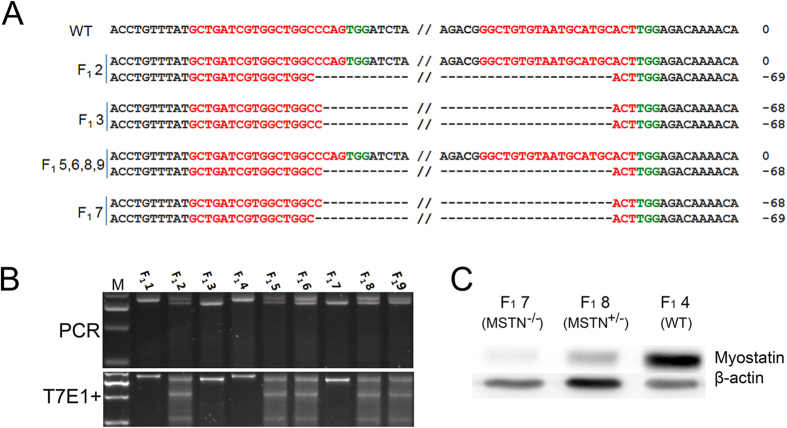
Heritability of the *MSTN* KO rabbit. (**A**) T-cloning and Sanger sequencing analyses of *MSTN* KO rabbits in F1. F_1_1–F_1_9 represent the F1 *MSTN* KO rabbits. The sequences of sgRNA are shown in red and the PAM sequences are presented in green. WT: wild type; deletions “−”. (**B**) The mutation detection of F1 rabbits by T7E1 cleavage assay. M, DL2000; F_1_1–F_1_9 represent the offspring pups used in this study. (**C**) Determination of *MSTN* protein in skeletal muscle by Western blot. Equal amounts of protein were used and the *β*-*actin* was used as reference control.

To determine whether the *MSTN* KO could be stably transmitted to the offspring, the female founder F_0_1–7 was mated with male rabbit F_0_1–5. T-cloning sequence analysis and T7E1 cleavage assay demonstrated that 7 out of 9 newborn F1 rabbits carried *MSTN* mutations. In these pups, the F_1_2, F_1_5, F_1_6, F_1_8, F_1_9 were monoallelic, while F_1_3, F_1_7 were biallelic *MSTN* KO rabbits (Fig. 3A,B). To investigate whether gene mutations abolished the *MSTN* protein translation, western blot analysis was carried out using the protein extracted from gluteus maximus tissue of *MSTN*^−/−^, *MSTN*^+/−^ and WT rabbits. As shown in Fig. 3C, the *MSTN* protein was significantly decreased in *MSTN*^−/−^ and *MSTN*^+/−^ rabbits, compared to their WT counterparts.

### Increased body weight and muscle mass in *MSTN* KO rabbits

**Figure 4:**
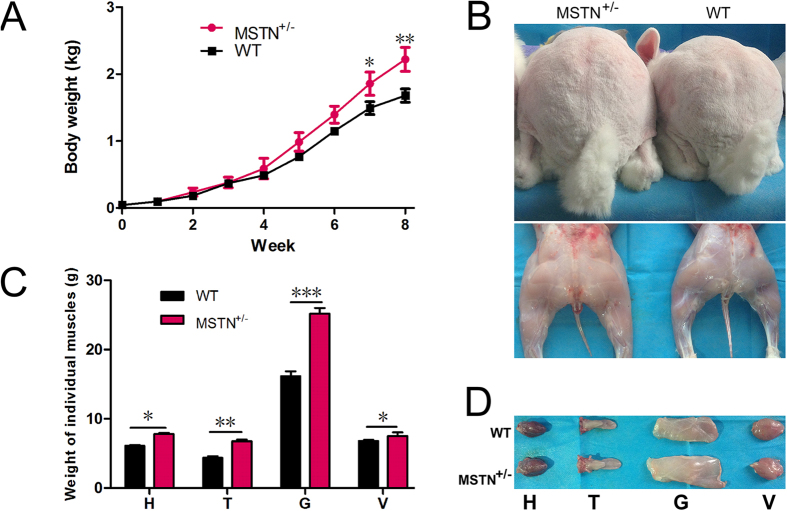
Increased body weight and muscle mass in *MSTN* KO rabbits. (**A**) The average body weight of *MSTN*^+/−^ and WT rabbits from F1 (n = 4). (**B**) Photos of *MSTN*^+/−^ (F_1_ 6) and WT (F_1_ 1) rabbits (2-month old) in F1. (**C**) The average weight of heart (H), tongue (T), gluteus maximus (G) and vastus lateralis (V) from *MSTN*^+/−^ and WT rabbits (n = 4). Data are expressed as mean ± SEM. *p < 0.05, **p < 0.01, ***p < 0.001, Student’s t test. (**D**) Representative of heart (H), tongue (T), gluteus maximus (G) and vastus lateralis (V) from *MSTN*^+/−^ and WT rabbits.

In order to investigate the differences in muscle development in *MSTN* KO rabbits, the body weight of *MSTN*^+/−^ and WT groups (n = 4) were recorded weekly. As shown in Fig. 4A, the body weight was not significantly different between the two groups during the first 6 weeks. However, the *MSTN*^+/−^ rabbits were obviously heavier than WT rabbits after 6 weeks of age. In addition, the typical double-muscled phenotype was also found in *MSTN*^+/−^ rabbits at 2 months of age (Fig. 4B).

To determine if the increase in body weight was caused by enlarged muscle mass in *MSTN* KO rabbits, the average weight of heart, tongue, gluteus maximus and vastus lateralis from *MSTN*^+/−^ and WT groups was determined at 2 months of age. As shown in Fig. 4C,D, no abnormal development was observed, while the average weight of individual muscles was dramatically increased in the *MSTN* KO rabbits, which compared to the WT counterparts.

### Hyperplasia and/or hypertrophy of muscle fibers in *MSTN* KO rabbits

**Figure 5:**
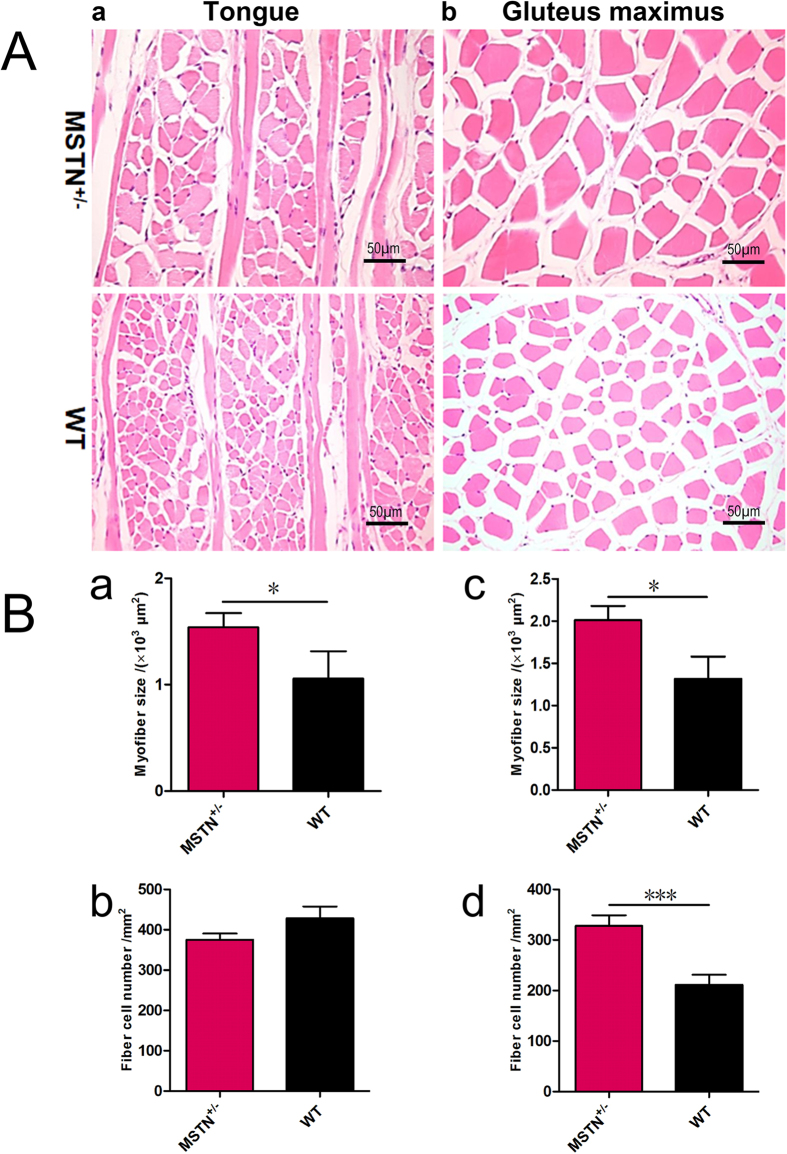
A hyperplasia and/or hypertrophy of muscle fibers was observed in *MSTN* KO rabbits. (**A**) H&E staining of the muscle fibers from tongue and gluteus maximus. (a) Cross and longitudinal section in tongue; (b) Cross section in gluteus maximus. (**B**) The statistical analysis of the fiber size and number in tongue and gluteus maximus from *MSTN*^+/−^ and WT rabbits (2-month old). Average fiber size and number in tongue (a,b); and in gluteus maximus (c,d). Data are expressed as mean ± SEM. *p < 0.05, **p < 0.01, ***p < 0.001, Student’s t test.

To determine whether the increase in muscle mass is due to hyperplasia and/or hypertrophy of muscle fibers, histological analysis was performed in the tongue and gluteus maximus from WT and *MSTN* KO rabbits (Fig. 5A). The results showed that the average size of tongue myofibers in *MSTN*^+/−^ rabbits (1539.20 ± 132.52 μm^2^, *p* < 0.05) was substantially larger than in WT rabbits (1055.81 ± 257.57 μm^2^) (Fig. 5B). Additionally, the average myofiber size in gluteus maximus was significantly increased in the *MSTN*^+/−^ (2011.01 ± 169.79 um^2^, *p* < 0.05) rabbits, compared to the WT rabbits (1318.51 ± 261.48um^2^). Furthermore, the average fiber density in gluteus maximus from the *MSTN*^+/−^ rabbits (327.79 ± 21.12, *p* < 0.001) was significantly higher than in the WT rabbits (211.37 ± 19.89) (Fig. 5B).

These results therefore suggest that the enlarged tongue in the *MSTN* KO rabbits is due to fiber hypertrophy. Whereas, it seems that the increased muscle mass of gluteus maximus in the *MSTN* KO rabbits is due to both fiber hyperplasia and hypertrophy.

## Discussion

In recent years, with the aim to study muscle development and increase meat production, *MSTN* has been modified in various species such as mice^14^, goats^15^, sheep^2^ and pigs^12^. In this study, we successfully generated *MSTN* KO rabbits by microinjection of Cas9/sgRNA into one-cell stage embryos, demonstrating that this system could be used to efficiently generate gene knockout rabbits. Moreover, fragment deletions were frequently detected between the two sgRNAs targeting sites in *MSTN* KO rabbits, suggesting that the dual sgRNA-directed CRISPR/Cas9 system could provide an efficient tool for gene knockout in the mammal genome^16^.

Off-target mutation has been frequently reported in Cas9-mediated gene editing system^17^,^18^,^19^, however, no off-target effect was detected in the *MSTN* KO rabbits. We believe that it might be due to the low concentration of Cas9/sgRNA, which degraded immediately after targeting the aimed gene. In addition, the strict match of seed sequences (8–12 bases close to PAM) is a critical factor for ensuring the site-specific cleavage of CRISPR/Cas9 system^20^. Furthermore, to avoid the off-target effect, two modified forms of Cas9, namely, D10ACas9^21^ and FokI-dCas9 (fCas9)^22^ can be used for gene editing in the future.

Although the increased muscle mass makes it attractive to create *MSTN* KO livestock, the calving difficulty induced by enlarged body size of fetus is a big problem in these *MSTN*-mutant animals^23^. It has been demonstrated that the *MSTN*-deficient animals exhibit disorders similar to large offspring syndrome (LOS)^8^,^24^. In our study, although the *MSTN* KO rabbits showed a typical double-muscled phenotype and a dramatically increased body weight, there was no significantly difference in body size and weight at birth compared to the WT controls. In addition, the *MSTN* KO rabbits appears normal, healthy, and without reproduction problems, indicating that *MSTN* KO rabbits generated by CRISPR/Cas9 system are suitable for the study of muscle development and related diseases.

Animals with *MSTN* mutations are characterized by increased muscle mass, which results from a combination of increased muscle fiber size and number^8^. Previous studies have demonstrated that the enlarged muscle is attributed to muscle fiber hyperplasia rather than hypertrophy in *MSTN*^−/−^ pigs^12^ and Belgian Blue cattle^25^. While both fiber hypertrophy and hyperplasia led to the increased muscle mass in *MSTN* KO mice^9^. In our study, both enlarged fiber size and increased fiber cell number were found in gluteus maximus, while the fiber hypertrophy was mainly responsible for the enlarged tongue in *MSTN*^+/−^ rabbits. Therefore we believe that the increased muscle phenotype in the *MSTN* KO rabbits is attributed to hyperplasia and/or hypertrophy of muscle fibers. Besides, the *MSTN*-regulated muscle proliferation and differentiation are also affected by expression levels of *MSTN* mRNA, the health state and the individual developmental stage of the organism.

In conclusion, the *MSTN* KO rabbits were efficiently generated by microinjection Cas9/sgRNA mixture into the pronuclear-stage embryos, and the typical double-muscled phenotype was obtained in the founder and offspring *MSTN* KO rabbits. These *MSTN* KO rabbits can be a promising tool for studying muscle development and improving economically important traits in livestock.

## Materials and Methods

### Ethics statement

All experiments involving animals were conducted according to the guidelines for the animal care and use of laboratory animals established by the Animal Care Center and Use Committee of Jilin University. All experimental protocols were approved by the Ethics Committee of Jilin University. New Zealand rabbits were housed under standard laboratory conditions of a 12-h light and 12-h dark cycle in individual cages, and were fed twice a day with commercial rabbit basic diet (Animal center of Jilin University) and water ad libitum. The sexually mature rabbits were used for embryo collection and embryo transfer recipients.

### Vector construction and *in vitro* transcription

The 3× FLAG-NLS-SpCas9-NLS vector (Addgene ID 48137) was linearized with *NotI* and *in vitro* transcribed to mRNA using the mMessage mMachine SP6 Kit (Ambion). Then the product was purified with the RNeasy Mini Kit (Qiagen) according to the manufacturer’s instructions.

To prepare the vector of *in vitro* transcription of sgRNA, the pair of complementary 20-nt DNA oligos were annealed to generate a double-stranded DNA fragment, and then sub-cloned into the *BbsI*-linearized pUC57-T7-gRNA vector (Addgene ID 51306). The sequence of two sgRNAs is shown in Supplementary Table S1. The PCR products for *in vitro* transcription of sgRNA were amplified using T7 primers (T7-F: 5′-GAAATTAATACGACTCACTATA-3′ and T7-R: 5′-AAAAAAAGCACCGA CTCGGTGCCAC-3′). Then *in vitro* transcription was performed using T7 RNA Synthesis Kit (Ambion) and the synthesized mRNA was purified using miRNeasy Mini Kit (Qiagen) according to the manufacturer’s instructions. The concentration and quality of the synthesized mRNA were determined by Nandrop 2000 and agarose gel electrophoresis, respectively.

### Zygote injection with Cas9/sgRNA

Zygotes were collected through surgical oviduct flushing from donors after superovulation treatment and natural mating, as described previously^5^. Rabbit embryos at the pronuclear stage (around 18–20 h post-mated) were transferred into oocyte manipulation medium, containing 9.5 g TCM-199, 0.05 g NaHCO3 (Sigma, S4019), 0.75 g Hepes (Sigma, H3784), 0.05 g penicillin, 0.06 g streptomycin, 1.755 g NaCl, 3.0 g BSA, and 1 L Milli Q H_2_O. A mixture of *in vitro* transcribed sgRNA (40 ng/μL) and Cas9 mRNA (180 ng/μL) was injected into the cytoplasm of pronuclear stage embryos. The injected embryos were transferred to embryo culture medium for 30–60 min culture at 38.5 °C, 5% carbon dioxide and humidity conditions. Then approximately 30–50 injected embryos were transferred into the oviduct of the recipient mother.

### Genotyping of *MSTN* mutation in embryos and pups

To test the mutation patterns, the injected embryos developed to blastocyst stage were collected and the genomic DNA was extracted with embryo lysis containing 1% NP40 at 50 °C for 20 minutes and 90 °C for 5 minutes in BIO-RAD PCR machine. The genomic DNA from ear punch tissues of newborn pups was isolated using the TIANamp Genomic DNA Kit (TIANGEN, Beijing, China). Then the target site was PCR amplified using the primers as follows: F 5′GGAGCAAGAGCCAATCATAGA 3′ and R 5′ TGAGGCTGTGAAGGCATAAG 3′. The PCR products were subjected to T7EI assay and T-cloning sequence.

The T7EI assay was performed as described previously^26^. Briefly, PCR products were purified with TIANgel Midi Purification Kit (TIANGEN, Beijing, China) and were denatured and annealed in NEBuffer 2 (NEB) using a thermocycler. Hybridized PCR products were digested with T7 endonuclease 1 (NEB, M0302L) for 30 minutes at 37 °C and subjected to 2% agarose gel electrophoresis.

### Off-target analysis

The POTS of the two sgRNAs were predicted using the CRISPR design tool (http://tools.genomeengineering.org). The top 5 POTS were selected for each sgRNA according to ranking scores. The PCR primers used in this study are shown in Supplementary Table S2. The PCR products were analyzed by T7EI assay and then cloned into the pGM-T (Tiangen, Beijing, China) for Sanger sequence.

### Western blotting and histology analysis

Samples from gluteus maximus tissue in *MSTN* KO and WT rabbits (euthanized at 2 months of age) of F1 generation were homogenized and lysed in RIPA buffer supplemented with 2.5 μL/mL protease inhibitor cocktail (Roche) on ice for 30 min. The protein concentrations were determined by Bradford method (Bio-Rad). 35 μg of protein sample was subjected to a 5% stacking/12% separating SDS-polyacrylamide gel. The antibodies used in this study include: anti-*MSTN* polyclonal antibody (Abcam) and goat anti-rabbit IgG conjugated with horseradish peroxidase (HRP) (Santa Cruz, USA). The *β*-*actin* antibody (Santa Cruz) was used as an internal control.

The tissues of tongue and gluteus maximus from *MSTN* KO and WT rabbits (euthanized at 2 months old) of F1 generation were fixed with 4% paraformaldehyde, then embedded in paraffin wax and slide sectioned. The sections were stained with hematoxylin and eosin (H&E) and analyzed by microscope (Nikon ts100).

### Body weight, carcass dissection, and sample collection

Four *MSTN*^+/−^ rabbits of F1 and four WT counterparts controls were bred under the same conditions and used in this study. All rabbits were weaned at 30 days of age and housed in individual cages of the same litter. The body weight was recorded weekly from 1–8 weeks. At 2 months of age, the rabbits were anesthetized with isofluorane, following the injection of beuthanasia (1 mL/4 kg). Then the carcasses were dissected and the heart, tongue, gluteus maximus and vastus lateralis of the *MSTN* KO and WT groups were weighed and recorded separately.

### Statistical analysis

Data was statistically analyzed by Graphpad prism software (T test) and a *p* value < 0.05 was considered statistically significant. *p < 0.05, **p < 0.01, ***p < 0.001.

## Additional Information

**How to cite this article**: Lv, Q. *et al*. Efficient Generation of *Myostatin* Gene Mutated Rabbit by CRISPR/Cas9. *Sci. Rep.* **6**, 25029; doi: 10.1038/srep25029 (2016).

## Supplementary Material

Supplementary Information

## Figures and Tables

**Table 1:** Generation of the *MSTN* KO rabbits via CRISPR/Cas9.

Recipients	gRNA/Cas9 mRNA(ng/μL)	Embryos transferred	Pregnancy	Pups obtained (% transferred)	Pups with mutations (% pups)	Bi- allelic modified (% pups)
1	40/180	40	YES	5(12.5%)	4(80%)	0(0%)
2	40/180	38	YES	4(10.5%)	2(50%)	0(0%)
3	40/180	40	YES	5(12.5%)	5(100%)	2(40%)
4	40/180	40	YES	6(15%)	5(83.3%)	4(66.7%)
