# Supplementary material for: Efficient Generation of Myostatin Gene Mutated Rabbit by CRISPR/Cas9
Source: Sci Rep. 2016 Apr 26;6:25029. doi: 10.1038/srep25029 (PMC4844959; doi:10.1038/srep25029)
Supplement: Supplementary Information [file srep25029-s1.docx]

**Supplementary Information**

**Efficient Generation of *Myostatin* Gene Mutated Rabbit by CRISPR/Cas9**

Qingyan Lv^#^, Lin Yuan^#^, Jichao Deng^#^, Mao Chen, Yong Wang, Jian Zeng, Zhanjun Li,

Liangxue Lai

**Inventory of Supplementary Information**

**1. Supplementary Figures**

**2. Supplementary Tables**

1. **Supplementary Figures**

**Supplementary Figure S1. Off target analysis of the *MSTN* KO rabbits.**

(A) T7E1 cleavage analysis of POTS (1-1 – 1-5 represent the five POTS for sgRNA1; 2-1 – 2-5 represent the five POTS for sgRNA2). M, DL2000;

(B) The chromatogram sequence analysis of POTS (1-1 – 1-5 represent the five POTS for sgRNA1; 2-1 – 2-5 represent the five POTS for sgRNA2). The 20bp of the POTS and the PAM are represented in shadow.


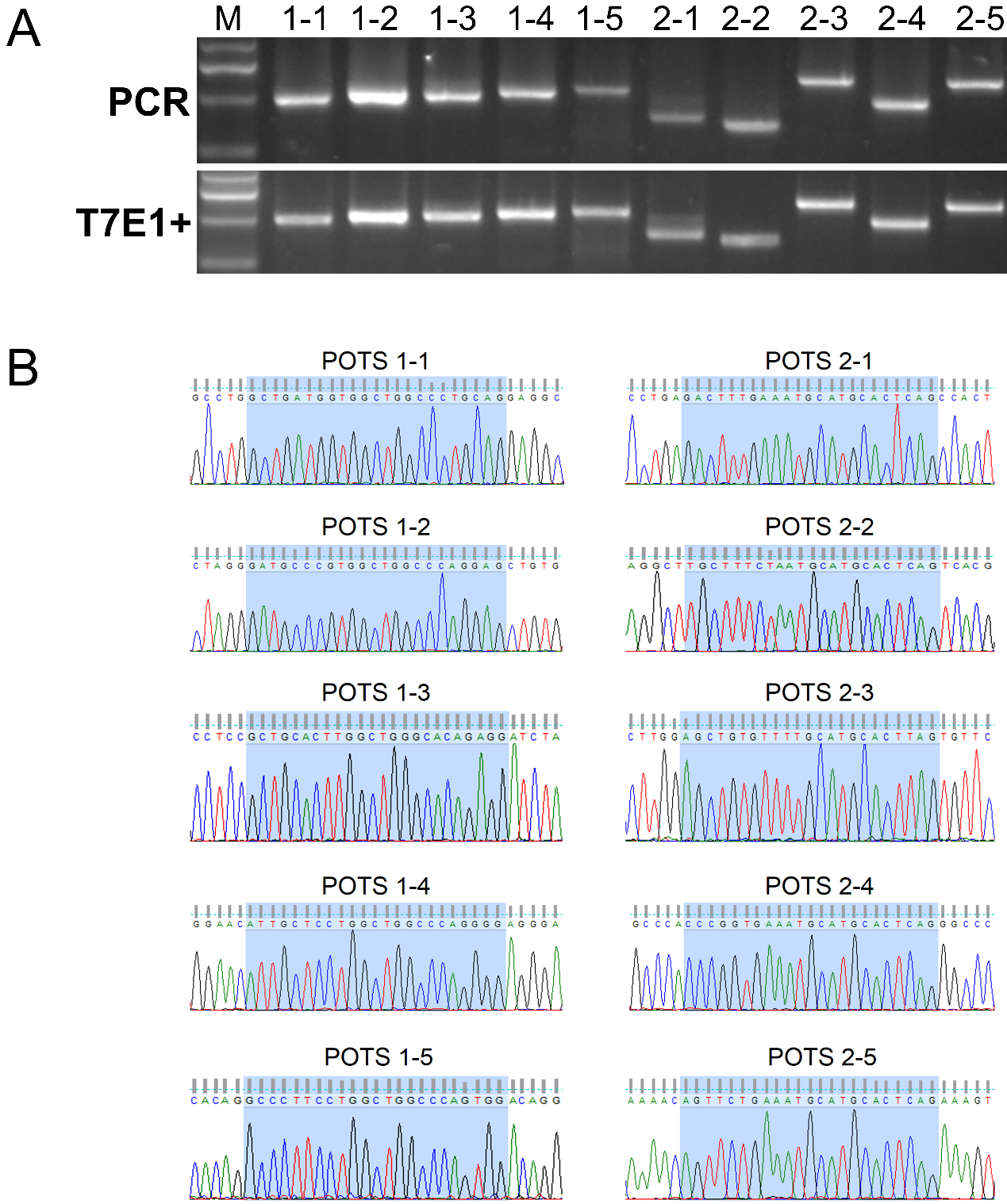


**Supplementary Figure S2. Histological longitudinal section of muscle fibers in tongue from**

***MSTN* KO and WT rabbits.**


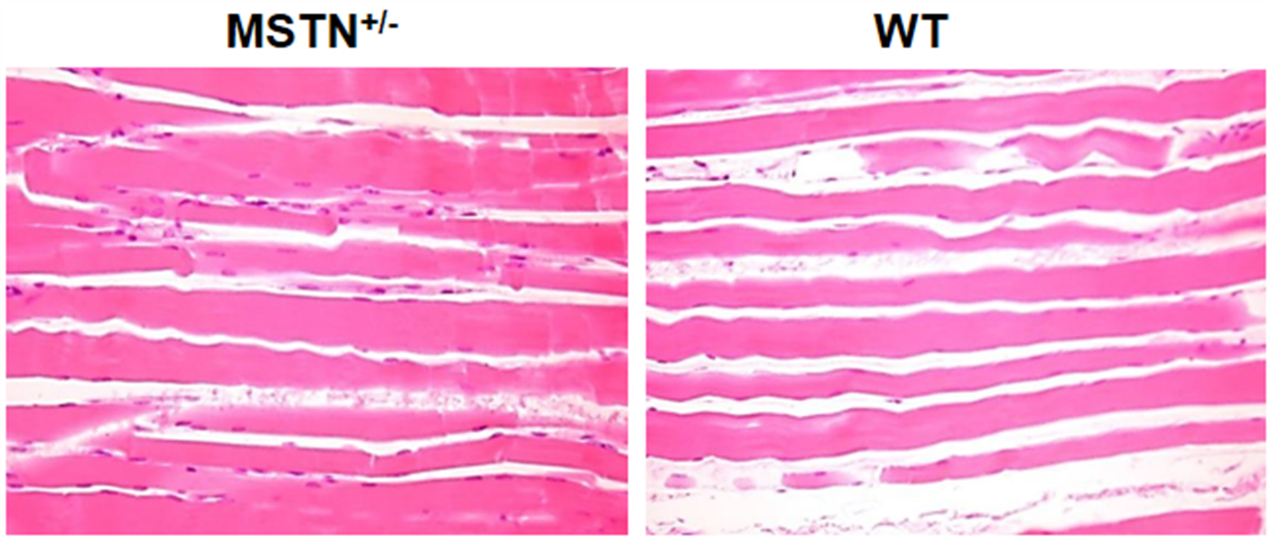


1. **Supplementary Tables**

**Supplementary Table S1. Two strands of oligonucletotides were used to construct the pUC57-sgRNA vector.**

| **sgRNA** | **Target site** | **PAM** | **Oligonucleotide1** | **Oligonucleotide2** |
| --- | --- | --- | --- | --- |
| *MSTN*-sgRNA1 | GCTGATCGTGGCTGGCCCAG | TGG | TAGGCTGATCGTGGCTGGCCCAG | AAACCTGGGCCAGCCACGATCAG |
| *MSTN*-sgRNA2 | GGCTGTGTAATGCATGCACT | TGG | TAGGCTGTGTAATGCATGCACT | AAACAGTGCATGCATTACACAG |

**Supplementary Table S2. PCR and sequencing of potential off-target loci.**

| **POTS** | **sequence** | **NO. of mismatch** | **Primer sequence**  **5’-3’** | **PCR prouduct lentgh** | **off-target** |
| --- | --- | --- | --- | --- | --- |
| **POTS1-1** | **GCTGATGGTGGCTGGCCCTGCAG** | **2** | **GGGCTGTGTGAGGAAGATAAG**  **CCCAGCTAGTAAGTGTCAAAGG** | **495** | **No** |
| **POTS1-2** | **GATGCCCGTGGCTGGCCCAGGAG** | **3** | **GCAGCTACTCCATACAGTTCTC**  **CGGTGACAGCCACATAGTT** | **532** | **No** |
| **POTS1-3** | **GCTGCACTTGGCTGGCCCAGAGG** | **3** | **TTTGAAACAAGATGGCCGATAAC**  **GAGTACAGAACTGGACGAAGAC** | **527** | **No** |
| **POTS1-4** | **ATTGCTCCTGGCTGGCCCAGGGG** | **4** | **TCTGAACAGGCGCAGAATAG**  **GGCACTGAAGACATCCTTTAGA** | **557** | **No** |
| **POTS1-5** | **GCCCTTCCTGGCTGGCCCAGTGG** | **4** | **GGCGCCACTGTTTCCTTAAA**  **GCAATAAGAGCCCGAACATCAG** | **583** | **No** |
| **POTS2-1** | **GACTTTGAAATGCATGCACTCAG** | **3** | **GGGCAGCGACAAGTTAGAG**  **TTTGCTCCATGTAGAGTCATCG** | **414** | **No** |
| **POTS2-2** | **TGCTTTCTAATGCATGCACTCAG** | **3** | **CTGAGCTAAGCCTGGTGAAATA**  **CTGGTAATAAGGGTCCACAGAC** | **384** | **No** |
| **POTS2-3** | **AGCTGTGTTTTGCATGCACTTAG** | **3** | **CACTTCAGCAGAGAGGAGTTAAG**  **AGGATGTTGGCTATTGGAGATG** | **680** | **No** |
| **POTS2-4** | **CCCGGTGAAATGCATGCACTCAG** | **4** | **CCAACCAGCTCCCTTTAATCT**  **CGCTTGTATCCACCCATTCT** | **518** | **No** |
| **POTS2-5** | **AGTTCTGAAATGCATGCACTCAG** | **4** | **CAGCACTCACTCCTTCCATTTA**  **CCTTCTCCGGCACATAAGTAAG** | **663** | **No** |

The letters in red are the mismatches compared with the sequence of sgRNA and the PAM are shown in green.
